# Phase I study of intermittent and chronomodulated oral therapy with capecitabine in patients with advanced and/or metastatic cancer

**DOI:** 10.1186/1471-2407-6-42

**Published:** 2006-02-24

**Authors:** Daniele Santini, Bruno Vincenzi, Gaia Schiavon, Annalisa La Cesa, Simona Gasparro, Angelo Vincenzi, Giuseppe Tonini

**Affiliations:** 1 Medical Oncology, University Campus Bio-Medico, Rome, Italy; 2 Engineering Course, University Campus Bio-Medico, Rome, Italy

## Abstract

**Background:**

The combination of capecitabine and gemcitabine at Fixed Dose Rate (FDR) has been demonstrated to be well tolerated, with apparent efficacy in patients with advanced cancers. FDR gemcitabine infusion leads to enhanced intracellular accumulation of drug and possible augmented clinical effect. The goals of this phase I study were to determine the maximum-tolerated dose (MTD) of chronomodulated capecitabine in patients with advanced cancer and to describe the dose-limiting toxicities (DLT), the safety profile of this way of administration.

**Methods:**

Patients with advanced solid tumours who had failed to response to standard therapy or for whom no standard therapy was available were elegible for this study. Capecitabine was administered orally according to following schedule: 1/4 of dose at 8:00 a.m.; 1/4 of dose at 6:00 p.m. and 1/2 of dose at 11:00 p.m. each day for 14 consecutive days, followed by a 7-day rest period.

**Results:**

All 27 patients enrolled onto the study were assessable for toxicity. The most common toxicities during the first two cycles of chemotherapy were fatigue, diarrhoea and hand foot syndrome (HFS). Only one out of the nine patients treated at capecitabine dose of 2,750 mg/m^2 ^met protocol-specified DLT criteria (fatigue grade 4). However, at these doses the majority of cycles of therapy were delivered without dose reduction or delay. No other episodes of DLT were observed at the same dose steps and at the lower dose steps of capecitabine (1,500/1,750/2,000/2,250/2,500 mg/m^2^). The dose of 2,750 mg/m^2 ^is recommended for further study. Tumor responses were observed in patients with metastatic breast and colorectal cancer.

**Conclusion:**

High doses of chronomodulated capecitabine can be administered with acceptable toxicity. The evidence of antitumor activity deserves further investigation in phase II combination chemotherapy studies.

## Background

Until recently, 5-fluorouracil (5-FU)-based therapy was the only drug regularly used for the palliation of patients with the most common types of cancer, such as colorectal, breast and stomach cancer, and it has been reported to have a limited impact on survival [1]. The overall treatment results remained unsatisfactory. It has only achieved 10% of objective responses, when given as a single agent for first-line treatment of metastatic disease [2,3]. The introduction into clinical practice of more effective treatment regimes with 5-FU, as the use of prolonged continuous infusion or the combination with other drugs, has led to an increase of responses [4-13]

Capecitabine, a carbamate derivative of 5'-DFUR, is absorbed through the gastrointestinal mucosa as an intact molecule and thereby it avoids the gastrointestinal toxicity associated with 5'DFUR. Infact, it is sequentially activated by carboxylesterase, cytidine deaminase and pyrimidine nucleoside phosphorylase. This cascade results in the formation of 5'-deoxy-5-fluorocytidine (5'-DFCR), 5'-deoxy-5-fluorouridine (5'-DFUR) and finally the intratumoral release of 5-FU [14]. Thymidilate phosphorylase (TP) catalyses the final step of capecitabine activation from the intermediate metabolite 5-deoxyfluorouracil to fluorouracil. 5-FU derived from the conversion acts on Thymidylate synthase (TS) and it is catalyzed by Dihydropyrimidine dehydrogenase (DPD). So its pharmacodynamic is similar to intravenous 5-FU [14].

In human colon cancer xenograft models, capecitabine was more effective at wider dose ranges than either 5-FU or 5'DFUR and showed a minor gastrointestinal toxicity [15]. Capecitabine has shown response rates in metastatic colorectal disease similar to infusional fluorouracil regimens at about 25%, with acceptable toxicity, and the convenience of an oral administration schedule [16].

In phase I and pharmacologic study by Mackean and collegues, capecitabine was administered in monotherapy twice daily as outpatient therapy, each cycle administered for 2 weeks followed by 1 week of rest. Thirty-four patients with advanced and/or metastatic solid tumors, all of whom except three patients were pretreated, were treated at dose levels from 502 to 3,514 mg/mq daily. They established that 3,000 mg/mq was not tolerated and they defined 2,510 mg/mq daily as the dose for phase II studies. Toxicities at this and lower dose levels were generally mild or moderate in nature and similar to those seen with continuous infusional 5-FU or 5'-DFUR treatment. The main drug-related toxicities were PPE and gastrointestinal, all of which were reversible and manageable with appropriate dose interruptions and modifications. The Authors recommended for further evaluation a dose of 2,510 mg/mq daily administered by the intermittent schedule [17].

We have recently conducted a phase II trial based on continuous infusion of oxaliplatin plus chronomodulated capecitabine in thirty-six 5-fluorouracil and irinotecan resistant advanced colorectal cancer patients [18]. Chronomodulated 5-FU clinical efficacy is maximum when administered in nocturnal hours with a concentration peak at 4:00 a.m. [19]. Capecitabine mimes continuous infusion of fluorouracil and is terminally converted in 5-FU. The thymidylate synthase (TP) is the enzyme responsible for this drug conversion and has not shown any specific circadian rhythm. The rational of capecitabine administration especially in nocturnal hours (1/4 dose at 6:00 p.m. and 2/4 dose at 11:00 p.m.), as performed in the mentioned study, was just based on the attempt to mime 5-FU chronomodulated infusion [18]. In particular, we administered oxaliplatin plus a chronomodulated schedule of capecitabine, such as 1750 mg/mq/die os (h 8.00 a.m. 25% of total dose; h 6.00 p.m. 25% of total dose; h 11.00 p.m. 50% of total dose), d 1–14 every 21 days. We demonstrated a high overall tumor growth control, a remarkable median time to progression (TTP) and overall survival (OS) and the good safety profile with diarrhoea (11.6%), nausea/vomiting (8.3%), neuropathy (8.3%), mucositis (8.3%), asthenia (16.7%) and hand-foot syndrome (5.5%), as most frequent related G3-4 adverse reactions.

Based on this phase II trial and on the above considerations, we conducted a phase I study aimed to determine the maximum-tolerated dose (MTD) and dose-limiting toxicities (DLTs) of capecitabine administered for two cycles of chronomodulated and intermittent therapy (two-weeks crhonomodulated oral therapy followed by 1 week of rest).

## Methods

### Patient selection

All patients had a baseline history and full physical examination with radiologic and laboratory evaluation. Patients with measurable or clinically assessable histologically confirmed advanced solid tumours who hade failed to response to standard therapy or for whom no standard therapy was available were elegible for this study. An age between 18 and 80 years, an Eastern Cooperative Oncology Group (ECOG) performance status ≤ 1 and a life expectance > 3 months were required. Bone marrow function requirements included an absolute neutrophil count ≥ 1500/mm^3^, a platelet count ≥ 100000/mm^3 ^and hemoglobin ≥ 10.0 g/100 ml. Preserved renal function (serum creatinine ≤ 1.6 mg/dL, normal creatinine clearance), hepatic function (total bilirubin ≤ 1.5 mg/dL, AST and ALT ≤ 2,5 times normal without hepatic metastasis and ≤ 4 times normal with hepatic metastasis; serum alkaline phosphatase < 2.5 times the upper limit of normal or < five times the upper limit of normal if liver metastases were present or < 10 times the upper limit of normal if bone metastases were present) and cardiac function were required.

Major exclusion criteria included cytotoxic or radiotherapy treatment within the previous 4 weeks (6 weeks if the previous therapy included a nitrosourea or mitomycin). Concomitant use of amiodarone, ketoconazole, itraconazole, diltiazem, verapamil, barbiturates, warfarin was not permitted. Pregnant or breast feeding woman or patients with uncontrolled severe disease were excluded. Patients with significant stomach, small intestine, liver, or kidney disease likely to affect drug absorption or metabolism were excluded from the study. Patients with history of coagulopathy or with nervous central system tumour or metastasis were excluded too. All patients were required to provide written informed consent prior to initiation of treatment, after a complete and opportune explanation. Local ethics committee approval was obtained. Women in fertile age had to be informed about risks incurring in case of pregnancy. The trial was conducted in accordance with the declaration of Helsinki. Patients were excluded if adequate follow-up was not possible (environmental or geographic difficulties, no compliance to undergo necessary clinical-instrumental investigations, etc.). The institution that granted ethical approval for the study was University Campus Bio-Medico of Rome. A specific informed consent was obtained from each participant before study entry.

### Study design

This is an open-label, single-center, nonrandomized, dose-escalating phase I study. All laboratory tests required to assess eligibility had to be completed within 7 days before start of treatment.

Capecitabine was administered orally according to following schedule: 1/4 of dose at 8:00 a.m.; 1/4 of dose at 6:00 p.m. and 1/2 of dose at 11:00 p.m. each day for 14 consecutive days (Days 1–14), followed by a 7-day rest period. Each treatment cycle is repeated every 21 days. Capecitabine was supplied as film-coated Xeloda tablets in two dosages strengths, 150-mg and 500-mg tablets administered not in fasting conditions, swallowed with water. No specific pre-medication for nausea and/or vomiting was provided (anticipated). A preventive protonic pump inhibitor (PPI) was advised to gastro-protective aim.

### Dose-escalation and definition of study end points

In phase I study of intermittent twice-daily oral therapy with capecitabine in patients with advanced and/or metastatic cancer, Mackean et al [17] used a starting dose level of 502 to 3,514 mg/mq daily, following a modified Fibonacci scheme. Since 3,000 mg/mq daily was not tolerated, they defined 2,510 mg/mq daily as the dose for phase II studies. Considering these results, the starting dose level in our phase I trial was 1750 mg/mq daily. The subsequent dose levels foreseen were: 2,000, 2,250, 2,500, 2,750, 3,000 mg/mq daily. In the absence of dose-limiting toxicities, only three patients were to be treated at the first two dose levels. At the third and subsequent dose levels, it was planned to treat at least six patients because it was anticipated that these dose levels may be therapeutic or associated with toxicity. Patients were seen weekly and toxicity was assessed by the National Cancer Institute of Canada Common Toxicity Criteria (NCI-CTC). For purposes of determining the MTD, only DLTs occurring during the first two cycles of therapy were considered. The MTD was defined as the dose level at which no more than one out of six patients experienced a DLT. Once this dose level was established, additional patients will be enrolled (maximum of 12) to gain additional experience with the combination. The MTD represents the dose recommended for further studies. DLTs were defined as any of the following: grade 4 neutropenia lasting at least 3 days or grade 3 or 4 neutropenia associated with fever ≥ 38.1°C; grade 4 thrombocytopenia lasting at least 3 days, grade 4 anemia; any grade 3 or 4 nonhematologic toxicity except alopecia, gastrointestinal toxicity, and palmar-plantar erythrodysesthesia (hand-foot syndrome); grade 3 or 4 nausea, vomiting, or mucositis; grade 3 and 4 diarrhea or a second occurrence of grade 2 diarrhea; grade 2 or 3 hand-foot syndrome not reduced to grade 1 before the start of cycle 2; delay of ≥ 14 days in initiating the second or the third cycle of therapy because of persistent toxicity of grade 2 or higher.

### Dose modifications

The capecitabine dose was interrupted or modified in spite of observed toxicity. There was no dose modification for grade 1 toxicity. For grade 2 toxicity that persisted despite symptomatic treatment, capecitabine was withheld until resolution to grade 0 or 1 and then restarted at the same dose. If the grade 2 toxicity recurred or any grade 3 toxicity, capecitabine was withheld until resolution to grade 0 or 1 and then restarted at the preceding dose. For grade 4 toxicity, capecitabine was discontinued unless considered by the investigator to be in patient's best interest, e.g. a responding patient, to continue at a lower dose level. In any case, patients who experienced DLT could be continued on treatment at a modified dose at the discretion of the treating physician if they seemed to be benefiting from the therapy.

### Pretreatment and follow-up studies

All patients had a baseline history and full physical examination with radiologic and laboratory evaluations. History, physical examination, and laboratory tests were repeated on day 1 of each cycle of therapy. Assessment of toxicity and hematology tests were performed weekly during each cycle of therapy [during and after the study period]. Response was assessed after two cycles of treatment (day 43) according to RECIST response criteria [20]. Responding patients or those with stable disease could continue treatment for a further four cycles of intermittent chronomodulated therapy with assessment of tumor response after four and six cycles (day 85 and 127, respectively). Patients who responded or had stable disease after six cycles could continue treatment at the discretion of the investigator.

## Results

### Patients

The characteristics of the 27 patients enrolled onto this study are listed in Table 1. The median age was 68 years (range, 49 to 88 years) and the median Eastern Cooperative Oncology Group (ECOG) performance status was 1 (range, 0 to 2). All of them except for six patients had previously received chemotherapy. 14 patients had previously received 5-fluorouracil-based chemotherapy. All patients completed the first two cycles of therapy and were, therefore, assessable for toxicity.

### Toxicity

The most common toxicities observed during the first two cycles of chemotherapy are listed in Table 2. No episodes of DLT were observed at capecitabine doses of 1,500/1,750/2,000/2,250/2,500 mg/m^2^. Only one patient treated at capecitabine dose of 2,750 mg/m^2^, met protocol-specified DLT criteria (grade 4 fatigue). 9 patients were treated at capecitabine dose of 2,750 mg/m^2 ^for a total of 49 cycles and median number of 5 cycles per patient (Table 3). At this dose step (2,750 mg/m^2^) we didn't observe any grade 3 or 4 toxicity (with the exception of one episode of grade 4 fatigue) during the first two cycles, but we observed two patients (22.2%) with grade 2 diarrhoea, two patients (22.2%) with grade 2 HFS, and two patients (22.2%) with grade 2 fatigue. All patients included in this dose step received the first two cycles at full doses without any dose reduction.

The hematologic and non-Hematologic toxicities cumulative over all cycles are reported in Table 4. At capecitabine dose level of 2,750 mg/m^2 ^we observed the following severe toxicities: 1 episode of diarrhoea grade 3 (11.1%), 1 episode of HFS grade 4 (11.1%) and 1 case of liver toxicity grade 3 (11.1%), all resolved after dose reduction. Hematologic toxicity was generally mild, with no patient experiencing clinically significant myelosuppression. Moreover, two patients required chemotherapy interruption because of severe or unresolved toxicity (respectively, 1 patient after three cycles for fatigue grade 3 and another patient after four cycles for cutaneous toxicity grade 3). At the same dose step 3 patients (33.3%) of the 9 included required (cycle 3 or higher) dose reduction or delay for toxicity. Despite the 2-week duration of dosing and the high dose level of capecitabine (2,750 mg/m^2^), few patients (3 patients, 33.3%) experienced clinically significant hand-foot syndrome (grade 2 or higher), no patients experienced febrile neutropenia or required platelet transfusion, and no patients reported episodes of severe nausea/vomiting or stomatitis (Table 4). The MTD was defined as the dose level at which no more than one of six patients experienced a DLT. Once this dose level was established, additional patients were enrolled (3 patients) to gain additional experience with the combination. Based on the occurrence of only one episode of DLT (fatigue grade 4) during the first two cycles and the ability to deliver the majority of successive cycles without dose modification or delay, we recommend doses of chronomodulated capecitabine of 2,750 mg/m^2 ^for further evaluation in phase II studies.

Although assessment of tumor response was not a primary objective of this study, patients were evaluated for tumor response after every two cycles of treatment. We observed 6 partial responses (PR) in breast cancer patients and 3 PR in colorectal cancer patients. The occurrence of responses in the colon cancer patient's subset is of particular note because all had previously received fluoropyrimidine-based therapy. Moreover, the median number of previous chemotherapeutic lines in responder patients was 2 (range, 0–4).

## Discussion

This study reports the first phase I experienced with chronomodulated administration of capecitabine. In the phase I studied by Mackean and collegues [17], capecitabine was administered in monotherapy twice daily, each cycle administered for 2 weeks followed by 1 week of rest. Thirty-four patients with advanced and/or metastatic solid tumors, all of whom except three patients were pretreated, were treated at dose levels from 502 to 3,514 mg/mq daily. They established that 3,000 mg/mq was not tolerated and they defined 2,510 mg/mq daily as the dose for phase II study. In this study the main drug-related toxicities were HFS and gastrointestinal. In the present study the rational of capecitabine administration especially in nocturnal hours (1/4 dose at 8 a.m., 1/4 dose at 6:00 p.m. and 2/4 dose at 11:00 p.m.) was just based on the attempt to mime 5-FU chronomodulated infusion. For this reason we decided to attempt a phase I study with the aim of establishing the MTD of chronomodulated capecitabine and the suggested dose for phase II studies. Interestingly, we observed that the chronomodulated way of capecitabine administration was safe and well tolerated in the majority of our patients. In fact, we reported only one episode of DLT in the nine patients included in the last dose step of capecitabine. For this reason, we recommended the dose of 2,750 mg/m^2^as the dose of capecitabine suggested for phase II studies. The main drug-related toxicities at dose step were HFS and gastrointestinal, all of which were reversible and manageable with appropriate dose interruptions and modifications. The high degree of tolerability of the intermittent chronomodulated schedule was also confirmed with a median number of five cycles (ie, 105 days) in patients who received 2,750 mg/m2 daily. Indeed, nine patients with a response or stable disease continued after the initial 9 weeks (three cycles) up to a maximum of 19 cycles (399 + days). The favorable toxicity profile of our regimen may have occurred because of the circadian organization of fluoropyrimidine metabolism, excretion and therapeutic targets. However, it could be also related to the thrice-daily schedule rather than the usual twice daily administration. We were encouraged to observe significant antitumor activity in this heavily pretreated patient population. Six breast cancer patients and 3 colorectal cancer patients obtained partial responses. Disease stabilization was also observed in 8 patients who, therefore, received multiple cycles of treatment. Phase II studies are now being planned for patients with breast cancer and colorectal cancer to further define the antitumor activity and tolerability of this regimen. Pharmacologic studies were not performed in this study, but it is likely that such studies would have contributed much information at this point in the development of this regimen. For this reason, a pharmacological study is strongly suggested. In conclusion, these results clearly highlight the need for new phase II trials to verify the real clinical impact of this new way of chronomodulated capecitabine administration in combination with other active drugs in solid tumors.

## Competing interests

The author(s) declare that they have no competing interests.

## Authors' contributions

DS conceived of the study, participated in its design and coordination and helped to draft the manuscript. BV participated in the design of the study and performed the statistical analysis. GS, AL, SG and AV participated in the data base compilation and in the conduction of the study. GT supervised all the steps of the study conduction. All authors read and approved the final manuscript.

## Pre-publication history

The pre-publication history for this paper can be accessed here:



## Figures and Tables

**Table 1:** Patient Characteristics

**Characteristics**	**No patients**
ECOG performance status	
0	10
1	11
2	6
Age, years	
Median	68
Range	49–88
Men/Women	9/18
Primary cancer	
Colorectal	8
Breast	12
Gastric	1
Pancreas	2
Others	4
Prior chemotherapy	
No of patients	21
Range per patient	1–4
Prior radiotherapy	12
Prior endocrine therapy	6
Prior 5-FU	14
No prior therapy	6

**Table 2:** Hematologic and Non-Hematologic Toxicities related to treatment in the first two cycles

		**No.patients who experienced toxicity, at maximum grade per patient, at each dose level, mg/mq/die**					
Toxicity	NCIC-CTC grade	1500	1750	2000	2250	2500	2750

Nausea/vomiting	0	2	3	2	2	5	4
	1	1	0	1	1	0	4
	2	0	0	0	0	1	1
	3*	0	0	0	0	0	0
	4*	0	0	0	0	0	0
Diarrhoea	0	2	3	3	2	3	3
	1	1	0	0	0	1	4
	2	0	0	0	1	2	2
	3*	0	0	0	0	0	0
	4*	0	0	0	0	0	0
Liver Toxicity	0	3	2	3	2	6	6
	1	0	1	0	1	0	3
	2	0	0	0	0	0	0
	3*	0	0	0	0	0	0
	4*	0	0	0	0	0	0
Stomatitis	0	3	3	2	2	5	6
	1	0	0	1	1	1	3
	2	0	0	0	0	0	0
	3*	0	0	0	0	0	0
	4*	0	0	0	0	0	0
HFS	0	3	2	3	2	4	4
	1	0	1	0	1	1	3
	2	0	0	0	0	1	2
	3*	0	0	0	0	0	0
	4*	0	0	0	0	0	0
Fatigue	0	2	2	1	0	3	2
	1	0	1	2	2	1	4
	2	1	0	0	1	2	2
	3*	0	0	0	0	0	0
	4*	0	0	0	0	0	**1**
Leuco-neutropenia	0	2	2	2	2	3	6
	1	0	1	0	0	1	1
	2	1	0	1	1	0	1
	3	0	0	0	0	1	1
	4*	0	0	0	0	0	0
Thrombocytopenia	0	2	3	2	3	5	6
	1	0	0	0	0	1	2
	2	1	0	1	0	0	1
	3	0	0	0	0	0	0
	4*	0	0	0	0	0	0
Anemia	0	1	2	1	2	3	7
	1	0	1	1	1	3	1
	2	2	0	1	0	0	1
	3	0	0	0	0	0	0
	4*	0	0	0	0	0	0

**Table 3:** Duration of treatment

Dose level [mg/mq/die]	No cycles administered [1 cycle = 21 days]	No. of patients	No. cycles per patient median [range]
1500	21	3	5 [2–14]
1750	33	3	10 [4–19]
2000	24	3	3 [2–19]
2250	24	3	8 [5–11]
2500	28	6	7 [2–10]
2750	49	9	5 [2–12]

**Table 4:** Hematologic and Non-Hematologic Toxicities cumulative over all cycles

		**No. patients who experienced toxicity, at maximum grade per patient, at each dose level, mg/mq/die**					
Toxicity	NCIC-CTC grade	1500	1750	2000	2250	2500	2750

Nausea/vomiting	0	2	2	2	2	4	4
	1	1	1	1	1	1	3
	2	0	0	0	0	1	2
	3*	0	0	0	0	0	0
	4*	0	0	0	0	0	0
Diarrhoea	0	2	3	3	0	3	3
	1	1	0	0	2	0	3
	2	0	0	0	1	2	2
	3*	0	0	0	0	**1**	**1**
	4*	0	0	0	0	0	0
Liver Toxicity	0	2	2	2	2	4	6
	1	0	1	0	0	1	1
	2	1	0	0	0	1	1
	3*	0	0	**1**	**1**	0	**1**
	4*	0	0	0	0	0	0
Stomatitis	0	3	2	1	2	5	4
	1	0	1	2	1	1	4
	2	0	0	0	0	0	1
	3*	0	0	0	0	0	0
	4*	0	0	0	0	0	0
HFS	0	3	2	3	1	2	3
	1	0	0	0	1	0	2
	2	0	0	0	1	3	3
	3*	0	**1**	0	0	1	0
	4*	0	0	0	0	0	**1**
Fatigue	0	2	0	1	0	2	2
	1	0	2	2	2	2	1
	2	1	1	0	1	2	5
	3*	0	0	0	0	0	0
	4*	0	0	0	0	0	**1**
Leuco-neutropenia	0	2	2	2	2	2	4
	1	0	0	0	0	3	1
	2	1	1	1	1	0	2
	3	0	0	0	0	1	2
	4*	0	0	0	0	0	0
Thrombocytopenia	0	2	2	2	3	4	6
	1	0	0	0	0	2	2
	2	1	0	1	0	0	1
	3	0	1	0	0	0	0
	4*	0	0	0	0	0	0
Anemia	0	1	1	1	0	1	5
	1	0	2	1	2	4	3
	2	2	0	1	1	1	1
	3	0	0	0	0	0	0
	4*	0	0	0	0	0	0
